# Supplementary material for: The discovery of novel noncoding RNAs in 50 bacterial genomes
Source: Nucleic Acids Res. 2024 Apr 22;52(9):5152–65. doi: 10.1093/nar/gkae248 (PMC11109978; doi:10.1093/nar/gkae248)
Supplement: gkae248_Supplemental_Files [file gkae248_supplemental_files.zip › Narunsky 50 Genomes RNA Motifs Files.zip!/Publication/69-E_rhusiopathiae/Figures/LRC-69-25-NC_015601_1_IGR_71_1339007_1339248.sto.pdf]

LRC-69-25-NC_015601_1_IGR_71_1339007_1339248.sto.r2r

5′-UCGR●●CUGU●UUYUU●AU●●UUCGYY●CAAAAUAUCCCCCCUUYC●CYUU●AUUUUAUU●●●●●U●AY●●A●●AU●C●A●ACAAR●UG●YUA●AUUGU●●●YUUYAUUGAUUACUYUGU●●UU●○YR●G●A●U●●Y●●RYRAAAAGGGAUUGACAAAAUUURAA●UCAAUYCUAGAAUAUAGGUGUUGGCACUC●YAY●●●●●RAGUGCCARAGGUCRAA●UAAAGGAGGAAACGCAU
